# Chronic Appendicitis, the Lesser-Known Form of Appendiceal Inflammation: A Case Report

**DOI:** 10.7759/cureus.19718

**Published:** 2021-11-18

**Authors:** Charles K Lee, Stephanie S Pelenyi, Orlando Fleites, Veronica Velez, Kayla L Alaimo, Darren N Ramcharan, Frederick Tiesenga

**Affiliations:** 1 Medicine, Saint James School of Medicine, Park Ridge, USA; 2 Surgery, West Suburban Medical Center, Oak Park, USA; 3 Anesthesia, Avalon School of Medicine, Willemstad, CUW; 4 General Surgery, West Suburban Medical Center, Oak Park, USA

**Keywords:** chronic abdominal pain, appendectomy, right lower quadrant pain, recurrent appendicitis, chronic appendicitis

## Abstract

Chronic appendicitis is a rare condition involving appendiceal inflammation as these conditions typically present acutely and are treated with appendectomy. However, in a small minority of patients, appendicitis can have a mild presentation and become recurrent or chronic appendicitis. Due to the acute nature and immediate treatment of patients presenting with typical symptoms of appendicitis, chronic appendicitis has been often overlooked and/or misdiagnosed.

We present a case in which a 50-year-old male presented with right lower quadrant (RLQ) pain of one-month duration. Computed tomography (CT) imaging showed evidence of lymph node enlargement near the patient’s appendix, raising suspicion of chronic appendicitis. The patient underwent a successful laparoscopic appendectomy.

## Introduction

Appendicitis is one of the most common abdominal surgical emergencies, affecting approximately 10% of the general population, most commonly in patients between 10 to 30 years of age [1]. The diagnosis of acute appendicitis is relatively simple; classically patients with the condition present with 48 hours of periumbilical pain that eventually localizes to the right iliac fossa (near McBurney’s point), associated with abdominal guarding, elevated white blood cell count, and anorexia [2]. Recurrent and chronic presentations of appendicitis are far less common than acute appendicitis; estimates, though scarce in the literature, suggest the incidence of chronic appendicitis is between 1% to 1.5% of all cases of appendicitis [2,3], and at least one reference suggests that up to 6.5% of appendicitis cases may involve recurrent or chronic episodes [4]. 

The pathophysiology of chronic appendicitis is not well-understood, but the condition is believed to be due to partial and/or transient obstruction of the appendix [2,5]. Traditionally chronic appendicitis has been defined as long-standing inflammation or fibrosis of the appendix manifested as right lower quadrant (RLQ) pain of more than 48 hours or intermittent RLQ pain [6]. Chronic and recurrent appendicitis are terms used interchangeably in the literature; however, at least one source differentiates between chronic and recurrent appendicitis, defining chronic appendicitis as appendicitis associated with three or more weeks of continuous RLQ pain and recurrent appendicitis as presenting with serial episodes of similar RLQ pain [7]. Patients with chronic appendicitis can present with recurring RLQ abdominal pain not associated with any febrile illness [8], or with symptoms that self-resolve [9]. These cases are milder than acute appendicitis, and their presentation is often non-specific or atypical for appendicitis [9]. As with acute appendicitis, chronic and recurrent appendicitis are treated with appendectomy [2,5-7,10]. Chronic appendicitis is not considered a surgical emergency [2]; however, it can go undiagnosed or be misdiagnosed and develop complications including perforation, abscess formation, peritonitis, and infertility, most of which require surgical intervention [1,2,8,11].

Here we present the case of a 50-year-old male (49 years old at the time of presentation) reporting one month of RLQ pain with abnormal computed tomography (CT) findings, which showed appendiceal enlargement with lymph node enlargement around the appendix suggestive of chronic appendicitis.

## Case presentation

Chief complaint

A 50-year-old male presented to the clinic with ongoing RLQ abdominal pain of one-month duration, associated with mild fever.

History of present illness

The patient was referred to the surgery clinic by his primary care physician (PCP) after experiencing ongoing RLQ pain and showing evidence of lymph node enlargement on CT imaging. At presentation, the patient reported a history of mild fever associated with RLQ pain. Two months prior to our encounter, the patient was started on an antibiotic regimen of levofloxacin with mild improvement in RLQ pain. Some mild pain symptoms continued. The patient also experienced pain while urinating and testicular pain approximately one month prior to the encounter. The patient denied any associated chest pain, shortness of breath, nausea/vomiting, or chills. The patient's heart rate and blood pressure were noted to be within normal limits.

Past medical history

The patient’s past medical history includes hypertension being treated with lisinopril, and diabetes mellitus being managed with metformin. Patient encounter records confirm that at some time between two and three months prior to presentation, the patient began taking levofloxacin daily. An occurence of painful rectal bleeding in 2015 prompted a colonoscopy, which revealed a colonic polyp and grade two hemorrhoids. The patient also suffered a left hand crush injury of the third and fourth digits in 2015.

Examination

At the time of the encounter, physical examination revealed RLQ tenderness, but was otherwise unremarkable. The patient’s heart rate, blood pressure, and other vital signs were within normal limits, and the patient was afebrile at the time of examination.

Investigations

**Figure 1:**
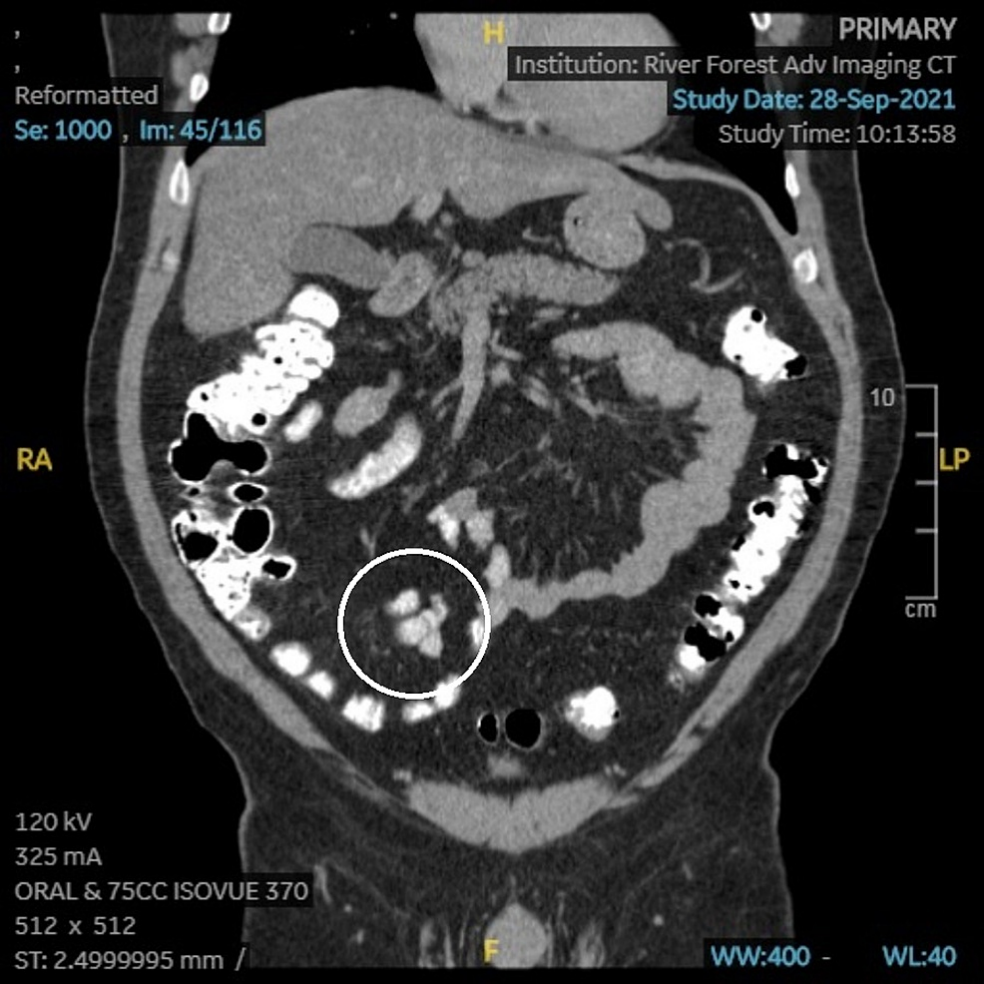
CT (coronal) image showing enlargement of the appendix and adjacent lymph nodes (circled).

**Figure 2:**
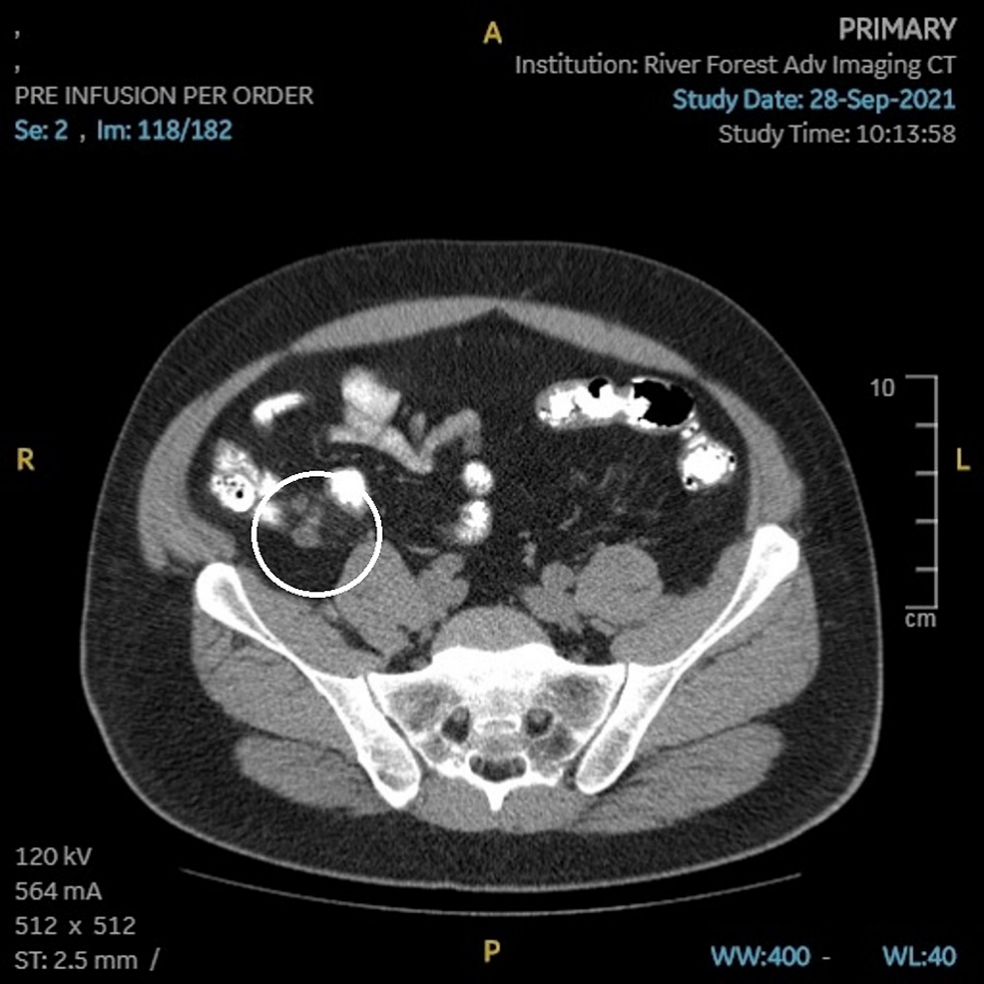
CT (transverse) image showing enlargement of the appendix and cluster of lymph nodes (circled).

**Figure 3:**
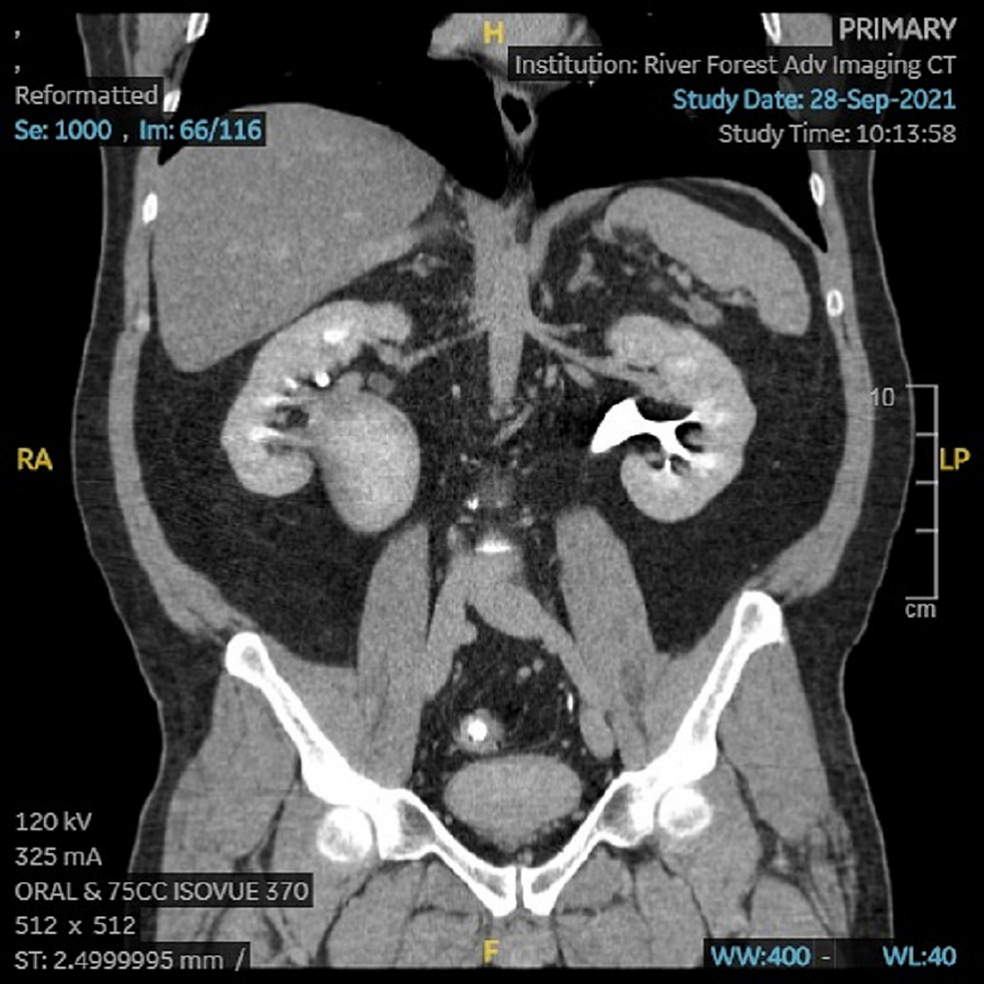
CT (coronal) image showing a very large right renal pelvis.

**Figure 4:**
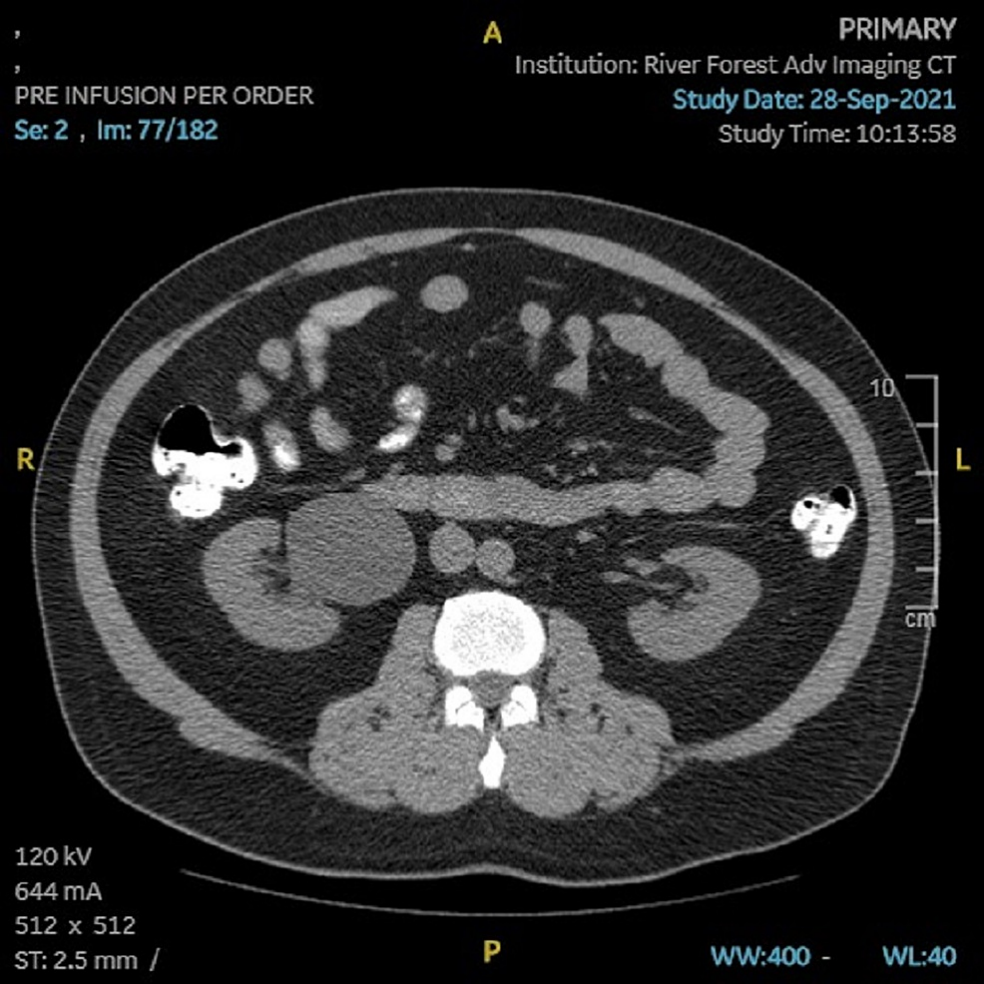
CT image (transverse) showing a very large right renal pelvis.

The patient’s PCP ordered CT imaging, which showed an appendiceal diameter on axial image of 8mm, and a cluster of prominent lymph nodes adjacent to the appendix, the largest of which was also 8mm (Figures 1, 2). Radiologic interpretation suggested these findings could be indicative of acute or chronic inflammation that may be an unusual presentation of chronic appendicitis. A dilated right extrarenal pelvis and proximal ureter with minimal right calyceal dilation were also seen (Figures 3, 4). In addition, a small non-obstructing stone in the right renal pelvis was seen, suggesting a chronic ureteropelvic junction stricture. Preoperative laboratory results showed a white blood cell count of 10.4x10^3 cells/µL.

Pre-operative diagnosis

Based on the patient’s history of present illness and associated investigations the preoperative diagnosis was chronic appendicitis.

Treatment

Laparoscopic appendectomy was performed on the patient. The appendix was identified and found to be chronically scarred and chronically inflamed in appearance. The mesoappendix was taken down with Sonicision, the base transected with Endo-GIA, and delivered from the peritoneal cavity in an Endobag and sent to pathology.

Post-operative diagnosis

The post-operative diagnosis was chronic appendicitis.

Outcome/progress

**Figure 5:**
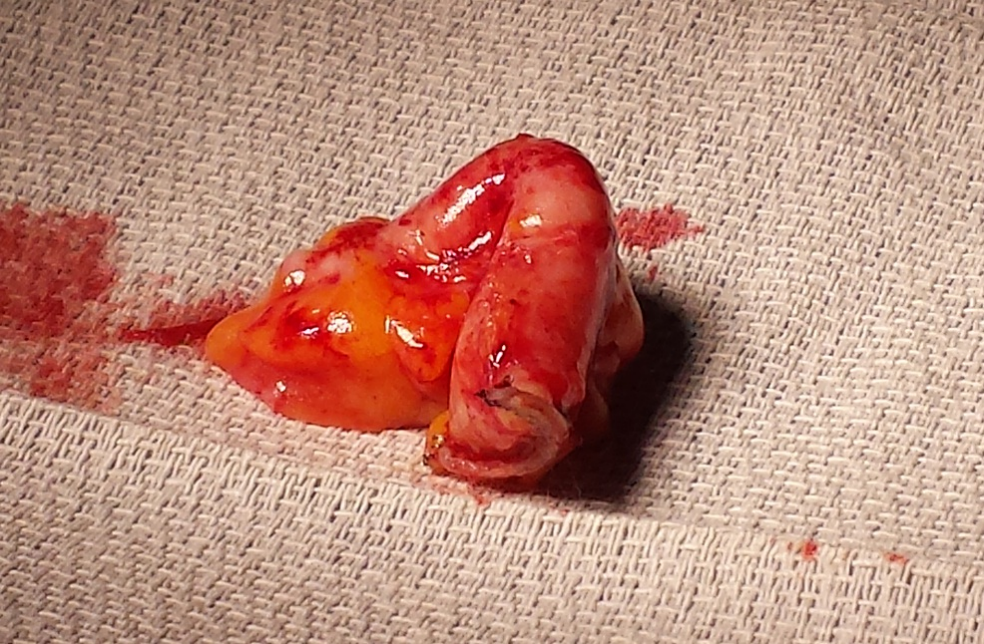
Image showing patient’s appendix after laparoscopic removal.

After recovering from anesthesia, the patient was discharged home on the same day as his procedure with no complaints and is currently being followed in an outpatient setting. The patient continues to report complete resolution of his symptoms. Postoperative histopathology report on the specimen taken from the patient (Figure 5) showed chronic lymphoplasmacytic inflammation with associated histiocytes and granulation tissue, further supporting the diagnosis of chronic appendicitis.

## Discussion

Even though traditional and updated definitions of chronic appendicitis exist, their application, and even the existence of chronic/recurrent appendicitis as a clinical entity, has been a matter of debate [6,7,10,12]. Mattei et al. (1994) concluded that “acute appendicitis can resolve spontaneously and recur repeatedly in the same individual,” and that in many cases of chronic or recurrent appendicitis, “they can be avoided by the accurate diagnosis and operative management of acute appendicitis” at initial presentation [12]. Several subsequent reports concur with this conclusion and agree that the primary treatment to resolve chronic/recurrent appendicitis is appendectomy [2,9,12], though at least one report suggests that this may not be necessary in younger patients [10].

Appendectomy as a treatment for appendicitis has been recorded as early as 1731 [13]. While prompt surgery is used to treat appendicitis complicated by events such as perforation, most uncomplicated cases of acute appendicitis are also treated surgically due to the potential to develop complications, especially after the publication of McBurney’s work at the end of the 19th century [14]. As most initial presentations of appendicitis are resolved with appendectomy, estimates of the incidence of chronic and/or recurrent appendicitis are few, suggesting the condition is relatively rare [2,3,9]. Some common features of chronic and recurrent appendicitis reported in the literature include administration of antibiotics for another preceding condition [2,5,7,10], milder symptoms than acute appendicitis [2,9,10,12], and often a normal white blood cell count [2,6,10] and/or afebrile presentation [2,5].

Computed tomography (CT) imaging has been suggested in the literature as early as 1998 as a primary method of identifying chronic and/or recurrent appendicitis [15]. Several case reports in the literature suggest that in addition to patient presentation, CT findings can help identify chronic and/or recurrent appendicitis with features such as minimal inflammatory stranding of the periappendiceal fat and thickening of the mid-portion of the appendix [2,6,9]. However, some other reports indicate that discovery of chronic and/or recurrent appendicitis was made intra-operatively [5,10] or that CT imaging was inconclusive [7].

Prior treatment with antibiotics is noted to have alleviated some of our patient’s appendicitis symptoms, such as RLQ pain, prior to our encounter. Our patient was found to have enlarged lymph nodes adjacent to an enlarged appendix on CT imaging, suggestive of chronic inflammation, which to our knowledge has not been reported anywhere else in the current literature. While chronic appendicitis was suspected based on this imaging and our patient’s presentation, this diagnosis was not confirmed until intraoperative examination revealed scarring and inflammation of the patient’s appendix consistent with a chronic process. The diagnosis of chronic appendicitis in our patient was further confirmed by histopathological findings from the surgical specimen, which were consistent with those reported in other cases of chronic appendicitis [9,10]. At follow-up our patient reported resolution of his RLQ pain, consistent with other reports in which appendectomy resolved pain associated with chronic appendicitis [2,5-7,10].

## Conclusions

Chronic appendicitis is a rare condition that is difficult to identify and is often initially misdiagnosed due to its atypical and/or milder presentation in comparison to acute appendicitis. However, like acute appendicitis, chronic appendicitis requires appropriate diagnosis and proper treatment, not only to resolve the milder but chronic symptoms of the patient, but also to avoid the possibility of perforation and other sequelae that can be caused by chronic appendicitis. CT imaging has been shown in a number of reports, as well as in our case, to greatly assist in the identification of chronic appendicitis.

Therefore, in afebrile patients presenting with mild right lower quadrant pain lasting several days to weeks, recent prior administration of antibiotics, and no prior history of appendectomy, chronic appendicitis should remain on the differential diagnosis. Imaging should include an abdominal CT scan, and if evidence confirms or is suggestive of chronic appendicitis, appendectomy is the treatment of choice.
